# Impact of Pulmonary Rehabilitation Services in Patients with Different Lung Diseases

**DOI:** 10.3390/jcm11020407

**Published:** 2022-01-14

**Authors:** Diana C. Sanchez-Ramirez

**Affiliations:** Department of Respiratory Therapy, College of Rehabilitation Sciences, University of Manitoba, Room 334-771 McDermot Ave, Winnipeg, MB R3E 0T6, Canada; diana.sanchez-ramirez@umanitoba.ca

**Keywords:** pulmonary rehabilitation, chronic obstructive pulmonary disease, asthma, bronchiectasis, interstitial lung diseases, restrictive lung diseases, lung cancer, pulmonary hypertension

## Abstract

Background: the effect of pulmonary rehabilitation (PR) services, beyond research contexts, on patients with lung diseases other than COPD requires further study. Objectives: to (i) assess the impact of a publicly funded PR on patients’ exercise capacity, self-efficacy, and health-related quality of life (HRQoL), and (ii) explore whether the effects vary across lung diseases. Methods: this retrospective pre–post study analyzed data from the Winnipeg Regional Health Authority PR program between 2016 and 2019. Results: 682 patients completed the full PR program. Pooled analyses found significant improvements in the patients’ exercise capacity (six-minute walk test (6MWT) (13.6%), fatigue (10.3%), and dyspnea (6.4%)), Self-Efficacy for Managing Chronic Disease 6-Item Scale (SEMCD6) (11.6%), and HRQoL (Clinical COPD Questionnaire (CCQ) (18.5%) and St George’s Respiratory Questionnaire (SGRQ) (10.9%)). The analyses conducted on sub-groups of patients with chronic obstructive pulmonary disease (COPD), asthma, bronchiectasis, interstitial lung diseases (ILDs), other restrictive lung diseases (e.g., obesity, pleural effusion, etc.), lung cancer, and pulmonary hypertension (PH) indicated that, except for patients with PH, all the patients improved in the 6MWT. Fatigue decreased in patients with COPD, ILDs, and other restrictive lung diseases. Dyspnea decreased in patients with COPD, asthma, and lung cancer. SEMCD6 scores increased in COPD, ILDs and PH patients. CCQ scores decreased in all lung diseases, except lung cancer and PH. SGRQ scores only decreased in patients with COPD. Conclusion: PR services had a significant impact on patients with different lung diseases. Therefore, publicly funded PR should be available as a critical component in the management of patients with these diseases.

## 1. Introduction

Respiratory diseases are among the top leading causes of disease, death, and disability globally. They account for more than 10% of all disability-adjusted life years worldwide [1]. Pulmonary rehabilitation (PR) is defined as a “comprehensive intervention based on a thorough patient assessment followed by patient-tailored therapies that include, but are not limited to, exercise training, education and behavior change, designed to improve the physical and psychological condition of people with chronic respiratory disease and to promote the long-term adherence to health-enhancing behaviors” [2]. This non-pharmacological intervention is known to decrease symptoms (dyspnea and fatigue), improve exercise tolerance and quality of life, reduce healthcare utilization, as well as increase physical activity among patients with COPD [3]. Emerging evidence also suggests that PR contributes towards improving outcomes among patients with interstitial lung disease [4], has small-to-moderate effects on exercise capacity in patients with lung cancer [5], and appears to be effective in patients with pulmonary hypertension [6]. However, the impact of PR on lung diseases other than COPD requires further study. Despite the multiple benefits identified [7,8], it has been estimated that less than 3% of people with chronic lung diseases accessed PR due to various reasons, including financial barriers [9]. Although some studies have explored the effect of PR in real-life settings [10,11], the impact of PR has been mainly reported in the context of research studies, and there is still a gap in the knowledge regarding the benefits of pulmonary rehabilitation services in the real world [8,12]. The American Thoracic Society (ATS) and the European Respiratory Society (ERS) indicated that more information is needed regarding the benefits of repeated courses of PR (especially for patients with chronic respiratory disorders other than COPD), suggested that healthcare professionals should conduct pragmatic “real-word” trials of PR, and recommended that further research should be undertaken to assess the impact of PR program funding sources on patient use [12]. Therefore, the objectives of this study were to (i) assess the impact of a regional publicly funded pulmonary rehabilitation program on patients’ exercise capacity, self-efficacy, and health-related quality of life, and (ii) explore whether effects vary across lung diseases.

## 2. Methods

### 2.1. Design

This retrospective pre–post study used anonymized data from patients with lung diseases collected routinely by the Winnipeg Regional Health Authority pulmonary rehabilitation program (chart review). This study was approved by the Health Research Ethics Board of the University of Manitoba (HS23753 (H2020:149)), and by Shared Health and the Winnipeg Regional Health Authority Research Access and Approval Committee (RAAC 2020-055).

#### Pulmonary Rehabilitation Program

The Winnipeg Regional Health Authority (WRHA) offers a publicly funded pulmonary rehabilitation program in three Winnipeg locations (Deer Lodge Centre, Misericordia Health Centre and Seven Oaks/Wellness Institute, Manitoba, Canada). This interdisciplinary collaborative program provides patient-centered care delivered by a team of healthcare professionals, including respirologists, physical therapists, respiratory therapists, pharmacists, kinesiologists, rehabilitation assistants, occupation therapists, dietitians, and social workers working with the patient’s primary healthcare provider. A physical therapist and a respiratory therapist conduct an intake assessment of patients, request specific consultation services (e.g., occupational therapy, dietitian, social work) when needed, deliver the exercise component, and guide the patients through the program. Once a month, the core healthcare team, composed of a respirologist, a physiotherapist, a respiratory therapist, and a pharmacist, meet to review the progression of the patients, and to explore the need for additional healthcare services (e.g., diagnostic tests, consult with other healthcare professionals) or changes in the action plan.

Patients are referred to the program by their primary healthcare provider (i.e., MD, nurse practitioner, specialist). The pulmonary rehabilitation program intake coordinator receives the referrals, processes them, and distributes them to one of the centers. At each center, patients are screened for inclusion and exclusion criteria. Inclusion criteria include the following: age ≥ 18 years; ability to attend an ambulatory community-based program; ability to understand education and exercise information; patients with pulmonary hypertension require approval from a respirologist; patients with COPD require confirmed diagnosis using spirometry or pulmonary function tests; other pulmonary diagnoses limiting exercise tolerance are considered on an individual basis (i.e., pulmonary fibrosis, bronchiectasis, restrictive lung diseases, asthma, etc.). Exclusion criteria include the following: inability to mobilize or care for self; severe, debilitating, and unmanaged pain issues; impaired ability to understand self-management and exercise education sessions; significant primary cardiac diseases (i.e., unstable angina or ischemia, acute pulmonary embolus or myocarditis, recent MI or heart surgery within the past 6 months, etc.); participant has attended the PR program once in the last two years (regardless of site). 

At each site, two groups of 8–10 patients complete the eight-week program twice a week at any given time (Tuesday/Thursday or Wednesday/Friday), starting 4 weeks apart to facilitate pre–post assessments. Each session (2 h) is split into two parts, half spent on education sessions delivered by various healthcare providers (Supplementary Table S1. Education session general outline), and the other half on an individualized exercise program. Every exercise session comprises breathing exercises, as well as warm-up, conditioning, and cool-down phases. The warm-up includes stretching, range-of-motion, and the beginning of low-intensity activities. The conditioning phase includes strengthening (upper and lower limbs) and aerobic exercises involving standard exercise equipment (i.e., dumbbells, treadmill, NuStep machine or cycle ergometer), and balance training. The cool-down phase includes a slow decrease in exercise intensity and stretching exercises. The duration and intensity of the activities are targeted to the capabilities and limitations of each patient. Heart rate and oxygen pulse oximetry are monitored throughout the session. 

### 2.2. Population of the Study

One thousand and eighty-five patients with chronic respiratory diseases participated in the Winnipeg Regional Health Authority pulmonary rehabilitation program offered in three Winnipeg locations between the calendar years 2016 and 2019. Sixty-three percent of the patients completed the full program (≥28 h). Patients who completed the pulmonary rehabilitation program were older and performed better at baseline than patients who did not complete it (Supplementary Table S2). 

### 2.3. Outcome Variables Were Assessed at the Beginning and End of the Pulmonary Rehabilitation Program

Exercise capacity. The six-minute walk test (6MWT) measures the distance (meters) that a patient can walk quickly on a flat, hard surface in a period of 6 min [13]. The minimal clinically important difference (MCID) for patients with COPD requires a mean improvement of 35 m (95% confidence interval 30–42 m) in the distance walked in the 6MWT at baseline [14]. Fatigue and perceived exertion were monitored before and after completing the 6MWT. Fatigue was assessed using a numerical rating scale (0 to 10, where 10 = “worst possible fatigue”) [15]; perceived exertion was assessed using the modified Borg scale (0 to 10, where 10 = “maximal: just like my hardest race”). This scale provides a good estimate of the actual heart rate during physical activity [16]. Dyspnea was assessed using the Medical Research Council (MRC) scale (0 to 5, where 5 = “too breathless to leave the house, or breathless when dressing”). This self-rating tool measures the degree of disability that breathlessness poses on a daily activity [17]. 

The Self-Efficacy for Managing Chronic Disease 6-Item Scale (SEMCD6) was used to assess the patients’ confidence performing certain activities to manage his/her condition. The SEMCD6 contains 6 items measured with a 10-step Likert scale, ranging from 1 “not at all confident” to 10 “totally confident”. It assesses the level of confidence required to manage fatigue, physical discomfort/pain, emotional distress, other symptoms/health problems, tasks/activities needed to manage health conditions, and strategies other than just taking medication [18,19]. 

Health-related quality of life (HRQoL) was assessed using the Clinical COPD Questionnaire (CCQ) and/or the St George’s Respiratory Questionnaire (SGRQ). The CCQ consists of 10 items (each score from 0 to 6), divided into three domains (symptoms, functional, and mental). The total score is calculated as the mean of the sum of all items, higher scores representing worse HRQoL [20,21]. The MCID of the CCQ score is −0.4 [22]. The SGRQ is a standardized questionnaire for measuring impaired health and perceived quality of life in airway diseases [23]. The questionnaire consists of 76 items divided into three parts (symptoms, activity limitation, and social and emotional impacts of the disease). Overall scores range from 0 to 100, with higher scores indicating poorer quality of life. The MCID of the SGRQ is −4 units [24]. 

### 2.4. Analysis

Descriptive statistics were used to present the characteristics of the participants of the pulmonary rehabilitation program, and baseline outcome variables. Percentages were used for categorical variables and means (standard deviations (SDs)) for continuous variables. Paired *t*-tests were used to explore mean differences in exercise capacity, self-efficacy and health-related quality of life before and after participation in the RP program. Cohen’s d was used to calculate effect size of the intervention (small (0.2), moderate (0.5), large (0.8)). Spearman correlations were used to examine relationships between changes in 6MWT, dyspnea, and self-efficacy with change in health-related quality of life after the pulmonary rehabilitation program. Statistical significance was set at *p*-values ≤ 0.05. All analyses were performed using IMB SPSS Statistic for Windows, version 24.0 (IBM Corp., Armonk, NY, USA). 

## 3. Results

### 3.1. Characteristics of the Population

Six hundred and eighty-two patients completed the pulmonary rehabilitation program, of which half (53.1%) were female. The mean age was 71.5 (SD 9.3) years. Five hundred and forty-one patients had chronic obstructive pulmonary disease (COPD), 89 had asthma, 33 had bronchiectasis, 127 had interstitial lung diseases (ILDs), 15 had other restrictive lung diseases (e.g., obesity, pleural effusion, diaphragm hemiparesis, rib involvement, etc.), 19 had lung cancer, and 12 had pulmonary hypertension (PH). Twenty-two percent of the patients had more than one coexisting lung disease (Table 1).

#### 3.1.1. Outcomes Improvement

In all the patients combined (Table 2), the distance walked on the 6MWT increased by 13.6%; fatigue and dyspnea scores decreased by 10.3% and 6.4%, respectively; self-efficacy improved by 11.7%; the total CCQ score decreased by 18.5%; the total SGRQ score decreased by 10.9%. Improvements in the 6MWT, CCQ, and SGRQ were greater than the MCID. Cohen’s effect size values suggested moderate-to-high practical significance of the PR intervention on the 6MWT (*d* = 0.73) and the CCQ *(d* = 0.60), and small-to-moderate practical significance on the SGRQ *(d* = 0.47), SEMCD6 (*d* = 0.32), dyspnea (*d* = 0.27), and fatigue (*d* = 0.11).

**Figure 1:**
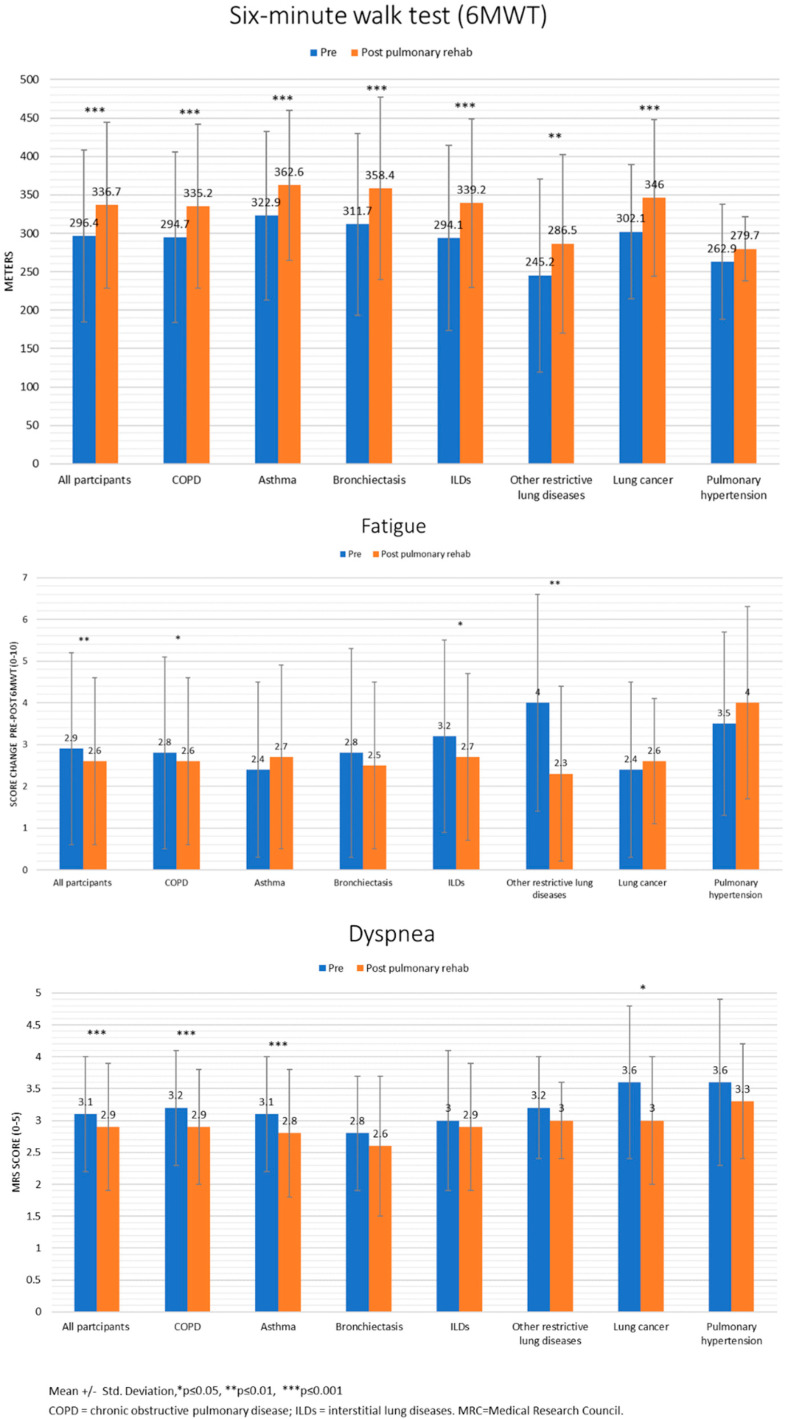
Exercise capacity pre-post Pulmonary Rehabilitation program.

**Figure 2:**
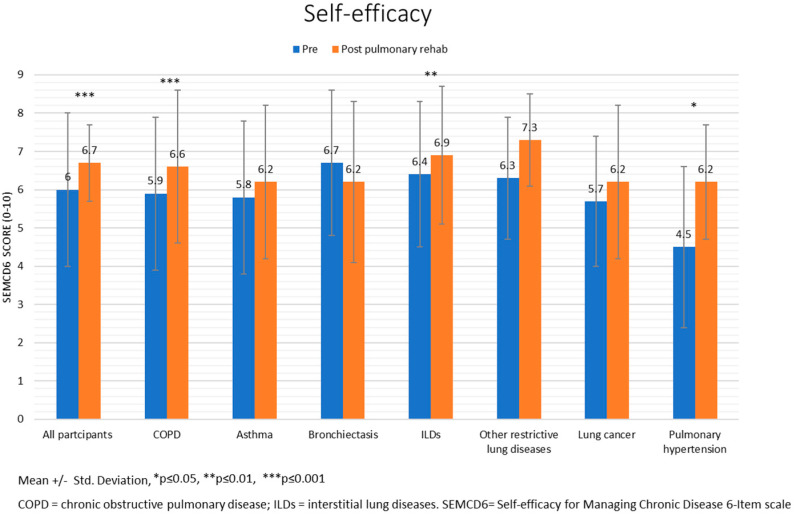
Self-efficacy pre-post Pulmonary Rehabilitation program.

**Figure 3:**
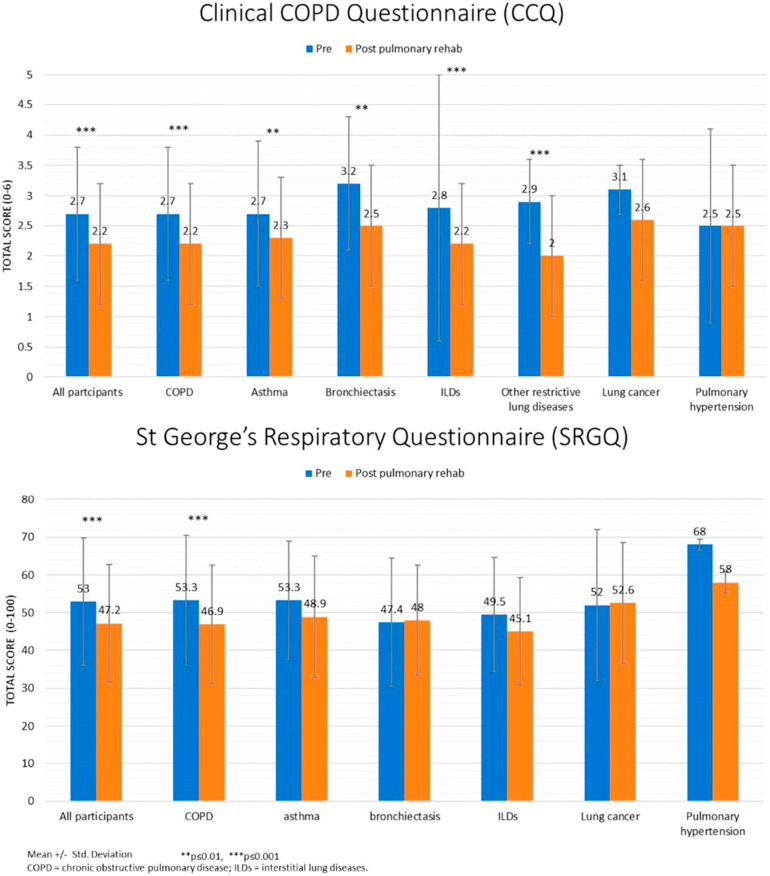
Health-related quality of life pre-post Pulmonary Rehabilitation program.

Figure 1, Figure 2 and Figure 3 present the results (mean ± standard deviation and *p*-values) of the sub-analyses conducted for lung diseases. Except for PH, patients with all lung diseases showed significant improvements in the 6MWT. Fatigue decreased in patients with COPD, ILDs, and other restrictive lung diseases. Dyspnea decreased in patients with COPD, asthma, and lung cancer. SEMCD6 scores increased in patients with COPD, ILDs, and PH; CCQ scores decreased in patients with COPD, asthma, bronchiectasis, ILDs, and other restrictive lung diseases; SGRQ scores only decreased in patients with COPD. 

#### 3.1.2. Outcomes Correlation

Greater distance walked during the 6MWT, decreased dyspnea, and increased self-efficacy were significantly correlated with better HRQoL in all the patients combined (Table 3). In patients with asthma, improved 6MWT was strongly correlated with better HRQoL. A weak correlation was found between decreased dyspnea and better HRQoL in patients with COPD. Increased self-efficacy was associated with better HRQoL in patients with COPD, asthma, and ILDs.

## 4. Discussion

Patients with chronic lung diseases who participated in pulmonary rehabilitation services offered by the publicly funded (WRHA) pulmonary rehabilitation program had statistically and clinically significant improvements in exercise capacity, self-efficacy, and health-related quality of life, which aligns with the evidence from previous research studies [7,8]. Although the benefits of pulmonary rehabilitation are well established, this intervention is often not included in the comprehensive care of patients with chronic lung diseases [25,26], and is frequently inaccessible to patients [27,28]. Some of the barriers to its access include lack of healthcare professional, payer, patient, and caregiver awareness of, and knowledge regarding, the processes and benefits of pulmonary rehabilitation; insufficient funding, limited resources, and inadequate allocation of health system reimbursement for pulmonary rehabilitation services [9,12]. 

Our sub-analyses for lung diseases identified a positive impact of pulmonary rehabilitation, although not to the same extent, in all patient groups. Patients with COPD experienced improvement in all outcomes studied, which is consistent with strong evidence supporting the benefits of pulmonary rehabilitation in patients with this lung disease [8]. Improvement in exercise capacity and HRQoL were also found in patients with asthma, bronchiectasis, ILDs, and other restrictive lung diseases, adding to the growing body of evidence on the impact of pulmonary rehabilitation on lung diseases other than COPD. The findings of this study align with previous evidence reporting a positive impact of pulmonary rehabilitation on exercise capacity and quality of life of patients with asthma [29] and ILDs [4]. The benefits of pulmonary rehabilitation have also been identified in patients with other restrictive lung diseases in conjunction with ILDs [30]; however, it seems that its effect has not been studied individually in this group of patients, which is one of the contributions of this study. There is sparse evidence regarding the impact of pulmonary rehabilitation on patients with bronchiectasis. A recent systematic review reported significant short-term improvements in exercise capacity and HRQoL; however, the review was hindered by a low number of trials (only 4), heterogeneity in the design, and a small number of patients (164 in total) [31]. Another systematic review (4 RCTs with a total of 262 participants) indicated that patients operated on for lung cancer may experience small-to-moderate improvements in exercise capacity and HRQoL [5]. In contrast, in the present study, patients with lung cancer had significant improvements in exercise capacity and dyspnea, but not in HRQoL. This discrepancy could be explained by the different tool used to measure HRQoL in previous studies (SF-36). In this study, patients with PH only improved in self-efficacy. Although evidence regarding the benefit of pulmonary rehabilitation in this group of patients is not conclusive [6], it is possible that the lack of improvement identified in the present study may be due to the small number of patients with this condition (n = 12). Further studies should explore potential reasons for the differences in the impact of pulmonary rehabilitation across lung diseases. Possible explanations may include a lack of specific content and appropriate assessment tools for each lung disease.

Health-related quality of life can be defined as “the gap between our expectations of health and our experience of it” [32]. A primary aim of the treatment of chronic diseases is to enhance quality of life by reducing the impact of the disease. However, the relationship between symptoms and exercise capacity, or functional limitation and quality of life, is neither simple nor direct. Therefore, we explored the association between HRQoL and other patient outcomes. Our results show that HRQoL correlated with increased 6MWT, indicating that 6MWT may be used in clinical practice to reflect the quality of life in all patients combined and in patients with asthma. HRQOL was also associated with decreased dyspnea in all the patients combined and in patients with COPD, which is consistent with a previous study that identified HRQoL to be adversely and independently associated with respiratory symptoms (dyspnea, wheeze, and cough), age, and female sex [33]. HRQoL was also significantly related to increased self-efficacy in all the patients combined, as well as in COPD, asthma, and ILDs patients. This aligns with previous evidence that identified self-efficacy as a predictor of improvement in health status and overall HRQoL in pulmonary rehabilitation [34].

## 5. Strengths and Limitations

The main strength of this study is the use of a large dataset, collected in real-life clinical services, to explore the effect of a publicly funded interdisciplinary and collaborative pulmonary rehabilitation program on the outcomes of patients with various chronic respiratory diseases. Several limitations of this study should be considered. First, this study used data routinely collected during patient care. Therefore, there were some gaps in terms of data completeness. Second, this study had no control group with which to compare the effects of an alternative intervention. Third, 22% of the patients had more than one co-existing lung disease, which may have influenced the results for individual lung diseases. However, in a real-word scenario, patients frequently have multiple comorbidities, sometimes even undiagnosed. Therefore, we still believe that the results of this study provide a valuable overview of the impact of pulmonary rehabilitation on the lung diseases studied. Furthermore, to avoid potential bias introduced by coexisting lung diseases in patients, the impact of the intervention was not compared between lung diseases. In addition, 37% of the patients did not complete the full pulmonary rehabilitation program (≥28 h). It is important to consider that 14% of the participants completed the program between 17 and 27 h, but because the data were collected by ranges, it was not possible to identify how many patients were close to completing the program. A previous study indicated that 31% of the patients referred to pulmonary rehabilitation did not attend the initial assessment, 10% did not enroll, and 17% did not complete the intervention [25]. Finally, patients who completed the pulmonary rehabilitation program were older and had better baseline outcomes than patients who did not complete it. Further studies should explore the causes of patient drop out and potential solutions. 

## 6. Conclusions

Pulmonary rehabilitation services provide statistically and clinically significant improvements in exercise capacity, self-efficacy, and health-related quality of life in patients with different lung diseases. Therefore, publicly funded pulmonary rehabilitation should be available as a critical component in the management of patients with chronic lung diseases. The results of this study contribute towards reducing the knowledge gap regarding the benefits of pulmonary rehabilitation services beyond research contexts, and may help raise awareness of the importance of facilitating access to this valuable, but currently still underutilized, intervention.

## Figures and Tables

**Table 1:** Characteristics of the participants who completed the pulmonary rehabilitation (PR) program 2016–2019 (n = 682).

Characteristic	n (%)
Females	362 (53.1)
Age, mean years (SD)	71.6 (9.3)
Oxygen users	122 (17.9)
Walking aid	
none	464 (72.3)
cane	32 (5.0)
4WW	143 (22.2)
Other ^#^	3 (0.5)
Location of PR program	
Deer lodge	243 (35.6%)
Misericordia Hospital	187 (27.4%)
Seven Oaks/Wellness Intitute	252 (37.0%)
Location of residence	
Outside Winnipeg region	74 (10.9)
Lung diseases *	
COPD	541 (79.5)
Asthma	89 (13.0)
Bronchiectasis	33 (4.8)
ILDs	127 (18.6)
Other restrictive lung diseases	15 (2.2)
Lung Cancer	19 (2.8)
Pulmonary hypertension	12 (1.8)

* Participants may have more than one respiratory condition. Abbreviations: 4WW = 4-wheel walker; ^#^ 3-wheel walker or oxygen tank; COPD = chronic obstructive pulmonary disease; ILDs = interstitial lung diseases including pulmonary fibrosis. Some percentages may not add up to 100% due to missing data.

**Table 2:** Change in outcomes for pre–post participation in the pulmonary rehabilitation program and effect size.

	All Participants			Change (95% CI)			*t*	df	Sig (2-Tailed)	Effect Size (d)
Outcomes	n	Pre Mean (SD)	Post Mean (SD)	Mean (SD)	Lower	Upper				
Exercise capacity										
6-min walk test (6-MWT) (meters)	633	296.4 (111.7)	336 (108.0)	40.3 (54.9)	44.5	36.0	26	632	<0.001	0.73
Fatigue (0–10 worse) Difference before–after 6MWT	602	2.9 (2.3)	2.6 (2.0)	0.3 (2.4)	0.07	0.46	2.7	601	0.007	0.11
Modified Borg Scale Difference before–after 6MWT (0–10 worse)	603	2.9 (2.1)	2.8 (1.8)	0.1 (2.2)	0.1	0.3	0.9	602	0.361	0.04
MRC dyspnea scale (0–5)	525	3.1 (0.9)	2.9 (1.0)	0.2 (0.8)	0.2	0.3	6.1	524	<0.001	0.27
Self-efficacy										
SEMCD6 (0–10 better)	428	6.0 (2.0)	6.7 (1.9)	0.6 (1.9)	0.8	0.4	6.7	427	<0.001	0.32
Health-related quality of life (HRQoL)										
CCQ Total score (0–6 worse)	389	2.7 (1.1)	2.2 (1.0)	0.5 (0.8)	0.4	0.6	11.7	388	<0.001	0.60
SGRQ Total score (0–100 worse)	152	53.0 (16.8)	47.2 (15.5)	5.8 (12.4)	3.8	7.8	5.7	151	<0.001	0.47

Paired *t*-tests were used to compare outcome values pre and post participation in the pulmonary rehabilitation program. MRC = Medical Research Council; CCQ = Clinical COPD Questionnaire; SEMCD6 = Self-Efficacy for Managing Chronic Disease 6-Item Scale; SGRQ = St George’s Respiratory Questionnaire. Cohen’s d was used to calculate effect size of the intervention (small (0.2), moderate (0.5), large (0.8)).

**Table 3:** Correlation of changes in 6-min walk test, dyspnea, and self-efficacy, with change in health-related quality of life, with regards to lung diseases.

	Lung Diseases	6-min Walk Test (m)	MRC Dyspnea Scale (0–5 Worse)	Self-Efficacy for Managing Chronic Disease 6-Item Scale (0–10 Better)
CCQ Total score (0–6 worse)	All combined	-	0.19 *p* = 0.001 (n = 318)	−0.30 *p* = <0.001 (n = 294)
	COPD	-	0.21 *p* = 0.001 (n = 275)	−0.31 *p* < 0.001 (n = 243)
	Asthma	-	-	−0.40 *p* = 0.01 (n = 41)
	ILDs	-	-	−0.29 *p* = 0.036 (n = 51)
SGRQ Total score (0–100 worse)	All combined	−0.22 *p* = 0.01 (n = 146)	0.19 *p* = 0.022 (n = 138)	−0.47 *p* < 0.001 (n = 93)
	COPD	-	0.19 *p* = 0.031 (n = 124)	−0.44 *p* < 0.001 (n = 78)
	Asthma	−0.61 *p* = 0.004 (n = 20)	-	-

Only statistically significant correlations are included in the table. Spearman’s correlation. MRC = Medical Research Council; CCQ = Clinical COPD Questionnaire; SGRQ = St George’s Respiratory Questionnaire; <0.4 = weak correlation; 0.40–0.59 = moderate correlation; 0.60–0.79 = strong correlation; ≥0.80 = very strong correlation.

## Data Availability

Data availability will be subject to approval by the Winnipeg Regional Health and Health Authority Research Access and Approval Committee.

## Supplementary Materials

The following supporting information can be downloaded at: https://www.mdpi.com/article/10.3390/jcm11020407/s1, Table S1: pulmonary rehabilitation education topics and order of presentations; Table S2: baseline outcomes of the participants by completion of the pulmonary rehabilitation program.
