# Supplementary material for: Impact of Pulmonary Rehabilitation Services in Patients with Different Lung Diseases
Source: J Clin Med. 2022 Jan 14;11(2):407. doi: 10.3390/jcm11020407 (PMC8780231; doi:10.3390/jcm11020407)
Supplement: Supplementary file 1 [file jcm-11-00407-s001.zip › jcm-1494790-supplementary.pdf]

Journal of Clinical Medicine

MDPI

Supplementary Materials

Table S1. Pulmonary Rehabilitation education topics and order of presentations.

Session	Topic	Delivered by
1	Intro to Pulmonary Rehab	Respiratory therapist
2	Managing shortness of breath & airway clearance	Physical therapist
3	Exercising well with chronic lung disease	Physical therapist
4	Risk factors & Triggers	Respiratory therapist
5	Action Plan	Respiratory therapist
6	Respiratory medication	Pharmacist
7	Medication delivery devices	Respiratory therapist
8	Healthy eating for people with chronic lung disease	Dietitian
9	Managing fatigue & energy conservation	Physical therapist
10	Strategies for coping with change & lost	Social worker
11	PC & ER visits, oxygen & travel	Respiratory therapist
12	Future planning	Social worker
13	Stress management	Physical therapist
14	Outcome measures	Physical therapist
15	Jeopardy Review	Respiratory therapist
16	Wrap-up & Tour of 55+	Physical therapist/ Respiratory therapist

Table S2. Baseline outcomes of the participants by completion of the pulmonary rehabilitation program.

		Completed the Program		
	n	No<br>n=403	Yes<br>n=682	*p*
Characteristics/baseline outcome				
Females	606	244 (60.5)	362 (53.1)	0.02
Males	479	159 (39.5)	320 (46.9)	
Age, mean years (SD)	1085	68.85 (11.2)	71.55 (9.3)	<0.001
Length of participation±				
1-5 hours (only assessment)	124	124 (30.8%)	-	-
6-16 hours	123	123 (30.5%)	-	
17-27 hours	156	156 (38.7%)	-	
28 hours or more (full program)	682	-	682 (100%)	
Oxygen users	199	77 (19.1)	122 (17.9)	0.61
Walking aid				
none	694	230 (64.2)	464 (72.3)	0.07
cane	56	24 (6.7)	32 (5.0)	
4WW	245	102 28.5)	143 22.3)	
Other#	5	2 (0.6)	3 (0.5)	
Location of residence				
Winnipeg region	958	350 (86.8)	608 (89.1)	0.25
Outside Winnipeg region	127	53 (13.2)	74 (10.9)	

Lung function baseline, mean (SD)				
FEV1, % predicted	1009	53.99 (22.5)	57.56 (23.0)	0.02
FVC, % predicted	1008	71.52 (22.2)	74.15 (20.1)	0.06
Lung diseases*				
COPD	870	328 (81.4)	542 (79.5)	0.44
Asthma	134	44 (10.9)	90 (13.2)	0.27
Bronchiectasis	57	26 (6.5)	31 (4.5)	0.17
ILD	172	46 (11.4)	126 (18.5)	<0.01
Other restrictive lung disease	22	8 (2.0)	15 (2.1)	0.93
Lung Cancer	26	8 (2.0%)	19 (2.6)	0.49
Pulmonary hypertension	15	4 (1.0)	12 (1.6)	0.39
6-minute walk test (6-MWT) (meters)	1028	279 (113.5)	294.5 (111.7)	0.04
Oxygen saturation before 6MWT (%)	988	94.6 (2.5)	94.9 (2.4)	0.16
Oxygen saturation after 6MWT (%)	987	91.1 (5.2)	90.9 (5.1)	0.69
Hearth rate before 6MWT (beats/min)	1002	79.3 (13.5)	77.6 (13.3	0.06
Hearth rate after 6MWT (beats/min)	988	100.9 (17.2)	98.9 (17.0)	0.07
Fatigue before 6MWT (0-10 worse)	1007	0.86 (1.3)	0.91 (1.4)	0.56
Fatigue after 6MWT (0-10 worse)	1000	3.94 (2.5)	3.83 (2.2)	0.48
Modified Borg Scale before 6MWT (0-10 worse)	1008	0.76 (1.1)	0.81 (1.1)	0.46
Modified Borg Scale after 6MWT (0-10 worse)	1001	3.70 (2.2)	3.78 (2.0)	0.58
MRC dyspnea scale (0-5)	790	3.42 (1.0)	3.17 (0.9)	<0.01
Sel-efficacy				
SEMCD6 (0-10 better)	651	5.32 (2.1)	6.12 (2.0)	<0.001
Health-related quality of life (HRQoL)				
CCQ Total score (0-6 worse)	626	2.98 (1.2)	2.72 (1.1)	<0.01
SGRQ Total score (0-100 worse)	228	57.45 (19.1)	52.81 (17.0)	0.07

*Participants may have more than one respiratory condition. 4WW= 4 wheels walker. #3 wheels walker or oxygen tank. ±Data recorded at intervals in the existing dataset. Abbreviations: 4WW = 4-wheel walker; # 3-wheel walker or oxygen tank; COPD = chronic obstructive pulmonary disease; ILDs = interstitial lung diseases including pulmonary fibrosis. Some percentages may not add up to 100% due to missing data.
